# Cost-effectiveness of apixaban versus low molecular weight heparin/vitamin k antagonist for the treatment of venous thromboembolism and the prevention of recurrences

**DOI:** 10.1186/s12913-017-1995-8

**Published:** 2017-01-23

**Authors:** Tereza Lanitis, Robert Leipold, Melissa Hamilton, Dale Rublee, Peter Quon, Chantelle Browne, Alexander T. Cohen

**Affiliations:** 1 Evidera, Metro Building 6th Floor, 1 Butterwick, London, W6 8DL UK; 2 Evidera, 7101 Wisconsin Avenue #1400, Bethesda, 20814 MD USA; 3 Bristol-Myers Squibb, 100 Nassau Park Blvd, Princeton, 08540 NJ USA; 4 Pfizer, 235 E 42nd St, New York, 10017 NY USA; 5 Guys and St Thomas’ NHS Foundation Trust, Westminster Bridge Road, London, SE1 7EH UK

**Keywords:** Venous thromboembolism, Apixaban, Vitamin K antagonists, Cost-effectiveness

## Abstract

**Background:**

Prior analyses beyond clinical trials are yet to evaluate the projected lifetime benefit of apixaban treatment compared to low-molecular-weight heparin (LMWH)/vitamin K antagonist (VKA) for treatment of venous thromboembolism (VTE) and prevention of recurrences. The objective of this study is to assess the cost-effectiveness of initial plus extended treatment with apixaban versus LMWH/VKA for either initial treatment only or initial plus extended treatment.

**Methods:**

A Markov cohort model was developed to evaluate the lifetime clinical and economic impact of treatment of VTE and prevention of recurrences with apixaban (starting at 10 mg BID for 1 week, then 5 mg BID for 6 months, then 2.5 mg BID for an additional 12 months) versus LMWH/VKA for 6 months and either no further treatment or extended treatment with VKA for an additional 12 months. Clinical event rates to inform the model were taken from the AMPLIFY and AMPLIFY-EXT trials and a network meta-analysis. Background mortality rates, costs, and utilities were obtained from published sources. The analysis was conducted from the perspective of the United Kingdom National Health Service. The evaluated outcomes included the number of events avoided in a 1000-patient cohort, total costs, life-years, quality-adjusted life-years (QALYs), and cost per QALY gained.

**Results:**

Initial plus extended treatment with apixaban was superior to both treatment durations of LMWH/VKA in reducing the number of bleeding events, and was superior to initial LMWH/VKA for 6 months followed by no therapy, in reducing VTE recurrences. Apixaban treatment was cost-effective compared to 6-month treatment with LMWH/VKA at an incremental cost-effectiveness ratio (ICER) of £6692 per QALY. When initial LMWH/VKA was followed by further VKA therapy for an additional 12 months (i.e., total treatment duration of 18 months), apixaban was cost-effective at an ICER of £8528 per QALY gained. Sensitivity analysis suggested these findings were robust over a wide range of inputs and scenarios for the model.

**Conclusions:**

In the UK, initial plus extended treatment with apixaban for treatment of VTE and prevention of recurrences appears to be economical and a clinically effective alternative to LMWH/VKA, whether used for initial or initial plus extended treatment.

**Electronic supplementary material:**

The online version of this article (doi:10.1186/s12913-017-1995-8) contains supplementary material, which is available to authorized users.

## Background

Deep vein thrombosis (DVT) and pulmonary embolism (PE) collectively constitute venous thromboembolism (VTE), the third most prevalent cardiovascular disease, [1] of which more than one million events occur annually among Europeans [2]. This clinical burden is reflected in the high total cost of VTE management, which in the United Kingdom (UK), for example, has been estimated at £640 million annually in direct and indirect costs (2004 estimate) [3]. A key determinant of this substantial economic burden is the need for medium- or long-term administration of anticoagulant therapy to treat acute VTE and help to prevent its recurrence. Specifically, guidelines recommend at least 3 months of anticoagulant treatment for VTE caused by a reversible risk factor, with the duration of treatment being long-term to at least 6 months in the case of unprovoked VTE or indefinitely in patients with certain risk factors (e.g., active cancer or the presence of other hypercoaguable conditions). Also, extended treatment durations are often chosen where patients have recurrent thromboses or a low risk of bleeding [4–7].

Until recently, the only available and recommended anticoagulants for use in VTE were heparins (e.g., low-molecular-weight heparin [LMWH]) or fondaparinux to initiate treatment, and vitamin K antagonists (VKAs) such as warfarin or acenocumerol to continue treatment and subsequent prevention of recurrence [8]. Use of these drugs is limited and complicated by their narrow therapeutic range and the burdensome requirement to monitor their anticoagulant effect by regularly measuring the international normalized ratio of the prothrombin time [9]. VKAs also have many drug-drug and drug-food interactions, which in turn affect international normalized ratio control and patients’ health-related quality of life [10].

The introduction of the drug apixaban, one of the new class of so-called direct oral anticoagulants (DOACs), could address many of the difficulties associated with VKA treatment, since by comparison, it exhibits non-inferiority in reducing VTE events, has a superior bleeding profile and does not impose a monitoring burden [11]. Evidence supporting this idea includes data from two double-blind randomised controlled trials that have studied apixaban. The AMPLIFY study was a non-inferiority trial that compared apixaban (10 mg twice a day [BID] for 7 days followed by 5 mg BID) to 7-day LMWH with concurrent initiation of VKA for 6 months in patients with acute VTE [12]. Apixaban provided a significant and clinically relevant reduction in major bleeding versus LWMH/VKA with a non-inferior reduction in recurrent VTE events [12]. The second trial, AMPLIFY-EXT, was in patients with VTE who had completed 6 to 12 months of anticoagulation therapy and in whom there was clinical equipoise as to whether to continue or stop therapy. It was a placebo-controlled superiority trial that assessed two doses of apixaban (2.5 mg and 5 mg BID) over a 12-month period and found that the drug significantly reduced the risk of recurrent VTE and VTE-related without increasing the rate of major or clinically relevant non-major bleeding [13].

A key question now is whether or not these clinical advantages of apixaban would be associated with any significant changes in health economic outcomes. This study aimed to address this evidence gap by using economic modelling to assess the cost-effectiveness of treatment and prevention of recurrences of VTE with apixaban versus LMWH/VKA. To do this, it compared the use of apixaban for an initial and long-term (6-month) treatment period plus a subsequent 12-month extended treatment period *(hereafter referred to as “initial plus extended apixaban”)* with the use of initial treatment with LMWH, in combination with long-term treatment for 6 months with VKA, as in the AMPLIFY and AMPLIFY-EXT studies (and *hereafter referred to as “initial LMWH/VKA)*. Apixaban was also compared to initial treatment with LMWH, long-term and extended for a total of 18 months with VKA *(hereafter referred to as “initial plus extended LMWH/VKA”)*.

## Methods

**Fig. 1:**
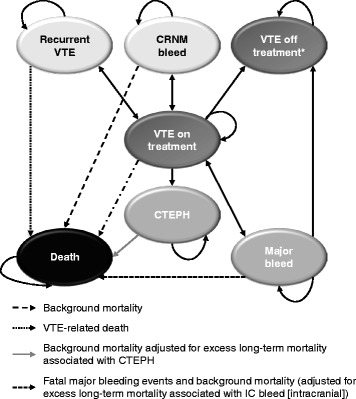
Model Diagram. *Patients in the off-treatment health state can experience the same events as patients in the on-treatment health states but at different risk levels. This diagram presents all potential health states that a patient may occupy while in the model. Arrows indicate potential health state transitions of patients. Dark Grey health states denote the treatment or originating health states. Patients are exposed to risks of recurrent VTE, CRNM bleed, CTEPH, major bleed, and death. The light grey health states (Recurrent VTE and CRNM bleed) are transient health states; that is, patients spend a temporary amount of time in this health state, and then return to their originating health state. CTEPH and Major bleeds are semi-absorbing health states; where patients do not experience any further events and remain in this health state until death. Major bleed is semi-absorbing if it is an IC bleed. If it is a non-IC bleed, it is a transient health state. PTS is modelled in the background and it is not a separate health state, but patients accrue expected costs and quality of life utility decrements associated with PTS while alive

The current study aimed to estimate the long-term clinical and economic outcomes for patients experiencing VTE, from the perspective of the UK National Health Service (NHS). To conduct this analysis, a Markov cohort approach was chosen as this approach has been used in previous evaluations for VTE and found to adequately capture the disease and consequences of treatment [14–16]. Such a model conceptualises the course of a disease by describing what might happen to a theoretical cohort of patients who spend time in various health ‘states’ that collectively represent the important clinical and economic consequences of the condition [17–19]. These states are mutually exclusive, and so a patient can be in only one of them at any given point. However, a patient can also move between health states through experiencing specific disease events, as long no more than one such transition occurs within each model ‘cycle’ – a fixed duration that recurs without interruption for as long as the model operates [17–19]. Crucially, the duration chosen for this cycle should reflect the disease being modelled: it needs be long enough to capture the full implications of a disease event but also sufficiently short that there would probably be only a single event in one cycle [17–19]. The chances that patients will move between health states during a cycle (the so-called ‘transition probabilities’) are a key design feature of the model [17–19]. With each cycle, the patients accrue (and the model computes) health care costs, life-years (LYs), and quality-adjusted life-years (QALYs) at rates dependent upon their health states. The model was developed in Microsoft Excel (Fig. 1), as this is a common platform for most users, and operated (using UK cost and outcome data to generate results, where possible). An example of the technical calculations included in the model is provided in Additional file 1.

In this study, all patients in the cohort were assumed to begin in the index VTE health state, having just experienced a VTE event and commenced anticoagulant treatment. Patients could move to other states or remain in the current state during each subsequent model cycle. Specifically, the states were either permanent (e.g., non-fatal intracranial bleed and chronic thromboembolic pulmonary hypertension [CTEPH]), in which case, patients remained in the health state without experiencing further events until death, or transient (e.g., recurrent DVT), such that patients spent a temporary period in the health state. The model used a 3-month cycle length, a duration consistent with those in previous VTE models [14, 15]. During each cycle, the cohort was subjected to competing risks of the following events: recurrent VTE, major bleeds, clinically relevant non-major (CRNM) bleeds, and death. Recurrent VTE events were classified as non-fatal recurrent PE, non-fatal recurrent DVT, or VTE-related death. Major bleeding events were classified as fatal and or non-fatal; and those that were non-fatal were further segregated between intracranial (IC) bleeds and non-IC bleeds. Patients experiencing a PE were at risk of CTEPH, whilst those experiencing a DVT were at risk of post-thrombotic syndrome (PTS), with risks for both events being independent of treatment. The model took into account only severe PTS, in view of published evidence suggesting that mild or moderate PTS had little effect on patients’ healthcare costs or self-assessment of their state of health [20, 21]. Patients were also at risk of treatment discontinuation, either as a result of major bleeding or because of adverse events unrelated to bleeding, in which case patients would move to ‘VTE off-treatment’ health states, where they would be exposed to the same events but at higher risk levels, in line with the fact that they were not receiving anticoagulant treatment. The model steps through multiple cycles until all patients from the initial cohort are dead.

Changes in treatment were modelled upon the occurrence of each event; but these treatment changes did not have an effect on subsequent transition risks in the model, only on costs and utilities. Patients experiencing a recurrent VTE event whilst on treatment were assumed to receive an additional 6 months of their current anticoagulation treatment. Patients currently off treatment were assumed to receive 6-month treatment with LMWH/VKA upon the occurrence of a recurrent VTE. Patients experiencing a non-fatal IC bleed were assumed to discontinue treatment permanently. Of the patients who experienced a non-fatal non-IC major bleed, 52.7% were assumed to discontinue treatment permanently, as calculated from secondary analysis of AMPLIFY using the number of treatment discontinuations due to major bleeding [12]. For the remainder of patients who experienced a non-fatal non-IC major bleed, it was assumed that treatment would be interrupted for 14 days before anticoagulant treatment was resumed. Patients experiencing a CRNM bleed were assumed to have their anticoagulant therapy interrupted for 2 days and then resumed thereafter for the remaining intended duration of treatment.

### Treatment

Treatment alternatives within the model mirrored those in the AMPLIFY and AMPLIFY-EXT trials. Treatment with apixaban was initiated at a dose of 10 mg BID for 1 week, then 5 mg BID long-term treatment for the remainder of the first 6 months, followed by extended treatment with 2.5 mg BID for an additional 12 months. This regimen was compared to LMWH initiated for at least 5 days, with dose-adjusted VKA therapy beginning concomitantly and continued for 6 months of long-term treatment followed either by no further extended treatment (as per AMPLIFY [12] and AMPLIFY-EXT [13]) or by an additional 12 months of extended treatment with dose-adjusted VKA.

The base-case analysis therefore compared the following:

- Initial plus extended treatment with apixaban for 18 months, versus initial treatment with LMWH/VKA for 6 months
- Initial plus extended treatment with apixaban for 18 months, versus initial plus extended treatment with LMWH/VKA for 18 months.

### Population

The population that required anticoagulation for the treatment and prevention of VTE recurrence consisted of 58.7% males and 41.3% females and entered the model with a mean age of 56.9 years [12]. It was assumed that 65.8% of patients, had experienced an initial DVT and 34.2% had experienced a PE [12]. These patient characteristics match those of the AMPLIFY clinical trial.

### Risk of clinical events

Table 1 presents the underlying time-dependent risks for each of the clinical events modelled for patients treated with apixaban. As suggested by clinical experts to the National Institute for Health and Care Excellence, there is no biological reason to believe that the treatment effects of anticoagulant therapy change over time, [22] therefore, justifying the application of constant relative treatment effects for VKA and placebo versus apixaban in the model, as presented in Table 2. Clinical event rates for recurrent VTE and VTE-related death, major bleeds, CRNM bleeds, and treatment discontinuation were obtained from secondary analysis of the AMPLIFY trial for the initial and long-term period (0–6 months) and the AMPLIFY-EXT trial for the extended period (beyond 6 months). Time-dependent risks of recurrent VTE and VTE-related death for untreated patients after 18 months (i.e., beyond the point to which patients were observed in the trials) was based on a prospective cohort study that followed patients with VTE over 10 years after treatment cessation [23]. The event rate for CTEPH was based on a prospective study that evaluated patients with PE treated with heparin infusion for 1 week followed by oral anticoagulation for 1 year. The study found that four of the 320 patients with PE developed CTEPH over a period of 2.1 years [24]. The risk of severe PTS was obtained from a prospective follow-up study of DVT patients who were treated with an initial course of LMWH followed by at least 3 months of oral anticoagulant therapy. The cumulative incidence of severe PTS was found to be 8.1% at 5 years, [25] consistent with more recently published estimates [26]. It was therefore assumed that there would be a constant risk of PTS of 8.1% in patients who had an index DVT, similar to the approach used in earlier models [16].

**Table 1:** Risks of clinical events over various treatment durations

	Apixaban	95% Confidence Interval (n)	Source
Recurrent VTE and VTE-related death risks per cycle			
0–3 months	1.71%	1.2–2.2%	^a^
3–6 months	0.48%	0.22–0.75%	^a^
6–9 months	0.48%	0.01–1.1%	^b^
9–12 months	0.59%	0.07–1.1%	^b^
12–15 months	0.12%	0.00–0.35%	^b^
15–18 months	0.36%	0.00–0.76%	^b^
Major bleed risk per cycle			^a^
0–3 months	0.41%	0.170.65%	^a^
3–6 months	0.15%	0.00–0.30%	^a^
Annual rate beyond 6 months	0.24%	0.00–0.57%	^b^
CRNM bleed risk per cycle			^a^
0–3 months	2.65%	2.04–3.26%	^a^
3–6 months	1.20%	0.78–1.61%	^a^
Annual rate beyond 6 months	3.00%	1.82–4.12%	^b^
Adverse event related discontinuation (not related to bleeding and VTE)			^a^
0–6 months	4.87%	4.05–5.68%	^a^
Annual rate beyond 6 months	6.67%	4.98–8.35%	^b^
Distribution of recurrent VTE events			
VTE-related death–on treatment	21.54%	(28)	^a^
Recurrent PE–on treatment	37.69%	(49)	^a^
Recurrent DVT–on treatment	40.77%	(53)	^a^
VTE-related death–off treatment	11.88%	(12)	^b^
Recurrent PE–off treatment	24.75%	(25)	^b^
Recurrent DVT–off treatment	63.37%	(64)	^b^
Distribution of major bleed events			
Fatal bleed–on treatment	13.46%		^a^
Non-fatal IC bleed–on treatment	13.97%		^a^
Non-fatal non-IC bleed–on treatment	86.03%		^a^
Fatal bleed–off treatment	13.46%		^b^
Non-fatal IC bleed–off treatment	13.97%		^b^
Non-fatal non-IC bleed–off treatment	86.03%		^b^
Chronic thromboembolic pulmonary hypertension (patients with PE) (rate per 2.1 years)	1.25%	0.03–2.46%	[24]
Post-thrombotic syndrome (patients with DVT) (% of patients experiencing per cycle)	8.10%	5.90–10.40%	[25]

^a^Data on file: Secondary Analysis of AMPLIFY (CV185-056) to Support Apixaban Cost Effectiveness Modeling for the Indication of Treatment of Deep Vein Thrombosis and Pulmonary Embolism in Venous Thromboembolisim (OR APIX 025). 2014

^b^Data on File: Secondary Analysis of AMPLIFY-EXT (CV185-057) to Support Apixaban Cost Effectiveness Modelling for the Extended Treatment of Deep Vein Thrombosis and Pulmonary Embolism in Venous Thromboembolisim (OR APIX 026). 2014

**Table 2:** Relative Treatment Effects for LMWH/VKA and Placebo versus Apixaban^§^

	Initial LMWH/VKA^a^	95% Confidence Interval	Extended Placebo^b^	95% Confidence Interval	Extended VKAs^c^	95% Confidence Interval
Relative risks						
Recurrent VTE and VTE-related death	1.18	0.83–1.66	5.33	3.02–9.40	0.42	0.16–1.06
Major bleed	3.26	1.84–5.79	2.07*	0.38–11.24	7.7	1.09–76.40
CRNM bleed	2.09	1.66–2.63	0.78	0.43–1.40	3.84	1.55–9.38
Other treatment discontinuation	1.07§	0.85–1.35§	-		1.33	0.75–1.87

*Absolute event rates for major bleed, apixaban: 0.0024, placebo: 0.0048

^§^The resulting risks for placebo and LMWH/VKA are detailed in the Additional file 2

Source:

^a^AMPLIFY [12]

^b^AMPLIFY-EXT [13]

^c^NMA (presented in the Additional file 2)

^§^Data on file: Secondary Analysis of AMPLIFY (CV185-056) to Support Apixaban Cost Effectiveness Modeling for the Indication of Treatment of Deep Vein Thrombosis and Pulmonary Embolism in Venous Thromboembolisim (OR APIX 025). 2014

Treatment effects for VKA compared with apixaban beyond the first 6 months in the form of relative risks were estimated by conducting a network meta-analysis (NMA) of clinical trials that had assessed extended anticoagulation treatment. Specifically, the NMA was conducted to examine the relative efficacy and safety of apixaban, rivaroxaban, dabigatran, aspirin, and warfarin (standard- and low-dose) in patients receiving extended treatment following initial treatment for an acute DVT and/or PE event. The effects for apixaban and no treatment were obtained from AMPLIFY-EXT, whilst those for VKA were obtained from the following trials: REMEDY, [27] LAFIT, [4] WODIT DVT, [28] and WODIT PE [29]. The data derived from the NMA are presented in the Additional file 2.

After the first 18 months in the model, the rates of major bleeding and CRNM bleeding were increased by a factor of 1.97 per decade of life to account for increased risk due to ageing [30]. These adjustments were not required before the first 18 months since rates for this initial period were based directly on the observed data from the AMPLIFY study and so would automatically reflect any added risk from ageing.

### Mortality

Background mortality was modelled based on age- and sex-specific UK life tables [31]. Hazard ratios reflecting the increased mortality associated with PE and DVT (excluding mortality due to bleeding and VTE recurrence, as these were explicitly modelled), CTEPH, and IC bleeds were applied to the background mortality rate to reflect the increased risk of death. These hazard ratios were taken from retrospective studies [32–34].

### Costs

Costs and sources are detailed in Table 3 and reflect 2011/2012 values. When prices for these years were not available, inflation rates were applied to the source data using the Pay & Prices index [35]. Anticoagulation costs were taken from the British National Formulary and electronic Market Information Tool [36, 37]. Administration and monitoring costs were based on a National Institute for Health and Care Excellence appraisal for rivaroxaban [16] and were calculated from the Personal Social Services Research Unit and NHS reference costs [35, 38]. It was assumed that six international normalized ratio monitoring visits would be required in the first 3 months followed by three visits every subsequent 3 months [39]. Event costs were separated into acute-care costs, which were applied at the time of the event, and maintenance costs. Maintenance costs were specified for two periods: the first 3 months after the acute event, and beyond the first 3 months following the event. Maintenance costs were applied only to those patients who experienced an IC bleed or CTEPH event. Patients experiencing all other events were exposed only to acute-care costs. These costs, along with those of treatment, were taken from NHS reference costs [38].

**Table 3:** Costs and Utilities

	Mean	Confidence Interval	Description	Source
Anticoagulant				
Daily cost of apixaban (initial period)^a^	£4.39			[36]
Daily cost of apixaban (prolonged and extended)^a^	£2.20			[36]
Daily cost of low-molecular-weight heparin (LMWH)/vitamin K antagonists (VKA) (initial period)^b^	£9.02			[36]
Daily cost of VKA (long-term and extended)^b^	£0.015			[36]
LMWH Administration				
One off cost for self-injection education (applied to all patients)	£17.50	£12.25–£22.75		[35]
Administration (for patients unable to self-inject)	£9.04	£6.33–£11.75		[35]
Proportion of patients who are able to self-inject	92%	64–100%		[16]
Monitoring				
Average INR monitoring cost			Assumption; NICE TA261 MS; NHS reference costs 2011/12, Outpatient procedures; 324 Anticoagulant Service	[38, 39]
⦁ First 3 months	£122.18			
⦁ Subsequent 3 months	£58.72			
VTE events				
DVT	£389.72	£272.80–£506.64	NICE TA261 MS; NICE CG92, NHS reference costs 2011/2012; QZ20Z, RA24Z, RA08A, RA60A, DAPF, 180	[38, 39]
PE or VTE-related death	£1340.41	£938.29–£1742.54	NICE TA261 MS; NICE CG92, NHS reference costs 2011/2012; DZ09A,DZ09B,DZ09C, RA24Z, RA08A, RA60A, DAPF, 180	[38, 39]
IC bleed (acute care)	£2760.57	£2017.43–£3252.62	NHS reference costs 2011/2012; AA23A; AA23B	[38]
IC bleed (maintenance)	£4387.76	£3685.78–£5107.61	NHS reference costs VC04Z	[38]
IC bleed (long-term)	£672.53	£473.01–£894.93		[54]
CTEPH (acute care)	£1888.23	£1379.02–£2225.48	NHS reference costs 2011/2012; AA23A; AA23B	[38]
CTEPH (long-term)	£4182.56	£2927.79–£5437.33		[43]
Non-IC major bleed	£1043.26	£785.90–£1192.06	NHS reference costs 2011/2012; FZ24A-C; FZ38D-F; FZ43A-C	[38]
CRNM bleed	£133.56	£113.86–£147.78	NHS reference costs 2011/2012; VB07Z	
PTS	£18.00	£12.60–£23.40		[35]
Utilities				
Utility estimates				
IC bleed (acute care)	0.3300	0.140–0.530	30 days	[41]
CTEPH (acute care)	0.6500	0.400–0.890	30 days	[55]
Utility decrements				
Apixaban	0.0020	0.000–0.0060	Whilst on treatment	[10]
LMWH/VKA	0.0130	0.000–0.0047	Whilst on treatment	[10]
DVT	0.1100	0.00–0.31	30 days	[41]
PE	0.3200	0.09–0.59	30 days	[41]
Non-IC bleed	0.3000	0.09–0.460	30 days	[41]
CRNM bleed	0.0054	0.00–0.0195	2 days	[42]
PTS	0.0700	0.00–0.24	Throughout	[20]

^a^4 × * 5 mg (induction), 2 × *5 mg (long-term), 2 × *2.5 mg (extended)

^b^300 mg multi-dose vial, price calculated based on patient weight of 84.6 kg of LMWH (induction), 1 × * 1 mg and 1 × * 5 mg of warfarin (long-term)

### Utilities

In order to generate QALYs, a key measure in the analysis, health state utilities were required as inputs for the model. Utilities represent an individual’s preferences for states of health, measured on a scale of 0 to 1, with 0 representing states of health equivalent to death, and a value of 1 indicating perfect health. On this basis, a utility for a given health state can be combined with the number of life-years a patient spends in that state to derive QALYs – an overall measure of both the duration and quality of life the patient experiences. Accordingly, the health states in the model vary with regard to their associated outcomes and utilities, and these are automatically updated by the model when patients experience events that occur and therefore move between health states. On entering the model all patients in the cohort had a baseline utility of 0.825 (0.003) applied, based upon a UK population-average score [40]. On the occurrence of a transient event (such as a PE, DVT, non-IC bleed, CRNM bleed or severe PTS) a utility decrement associated with that event was subtracted from the baseline utility, to reflect the negative impact of the episode on the patient’s health-related quality of life [10, 20, 41, 42]. The pre-specified durations for which these events were assumed to impair health-related quality of life were based on expert opinion and published literature [43, 44]. On the occurrence of a permanent event (such as an intracranial bleed or CTEPH), the patient’s utility value was updated and applied for the rest of their lifetime. Patients who moved to the death state were assigned a utility of 0. In addition to event-related decrements, it was assumed that anticoagulation use would have a negative impact on the health-related quality of life of patients who were on treatment. Utility values and their sources are detailed in Table 3.

Health and cost outcomes were discounted at 3.5% per annum and the model had a lifetime time horizon [45].

### Analyses

The base-model measured benefits in terms of LYs, QALYs, and costs accumulated over the time horizon of the model. The relative clinical benefit of apixaban versus LMWH/VKA was assessed using the incremental cost-effectiveness ratio (ICER), to ascertain whether the benefit gained was obtained at a cost less than the UK payers’ usual willingness-to-pay threshold of £20,000 per QALY [45].

One-way sensitivity analyses were conducted to evaluate the robustness of the model results and conclusions in relation to uncertainties in key model inputs, and to assess how the model outcomes varied in relation to changes in model parameters. Probabilistic sensitivity analysis was conducted to account for the statistical uncertainty in parameter estimates. Each parameter included in the model was assigned a probability distribution according to its mean value and 95% confidence interval. In each probabilistic sensitivity analysis run, a value was sampled from the probability distribution of each parameter and used to generate a corresponding pair of incremental QALYs and incremental costs. The probabilistic sensitivity analysis was run for over 2000 simulations, thereby producing 2000 pairs of incremental QALYs and costs that took into account the distribution of potential values in the input parameter as dictated by their confidence intervals. These pairs of QALYs and costs were then used to determine the probability of cost-effectiveness.

In addition to the above, and due to the uncertainty about the duration of treatment, a scenario analysis was conducted to assess how the cost-effectiveness of apixaban changed when treatment was extended over lifetime. To do this, the duration of treatment in the model was varied for initial plus extended apixaban from 18 months to indefinite treatment and compared to the following:

- Initial treatment with LMWH/VKA for 6 months without altering the duration of treatment.
- Initial plus extended treatment with LMWH/VKA over an indefinite duration.

## Results

### Base-case analysis (Table 4)

The evaluation in the current study predicted that, compared to 6 months of LMWH/VKA followed by no treatment, initial plus extended treatment with apixaban (as studied in the AMPLIFY trials) would lead to 62 fewer recurrent VTEs and VTE-related deaths, 13 fewer major bleeds, and 28 fewer CRNM bleeds over the lifetimes of 1000 treated patients. Compared to initial plus extended treatment with LMWH/VKA, apixaban was projected to lead to 26 fewer major bleeds and 111 fewer CRNM bleeds but, also, an additional 6 recurrent VTEs and VTE-related deaths. The increase in the number of recurrent events was attributed to a greater treatment effect for VKA in the extended period.

**Table 4:** Deterministic Results

	Apixaban	LMWH/VKA/Placebo	Difference	Apixaban	LMWH/VKA	Difference
Number of events (total population)						
Recurrent VTE and VTE-related death	514	577	−62	514	508	6
*VTE-related death*	*63*	*71*	*−8*	*63*	*63*	*0*
* Recurrent PE*	*130*	*146*	*−16*	*130*	*129*	*1*
*Recurrent DVT*	*321*	*359*	*−39*	*321*	*316*	*5*
Major bleeds	123	136	−13	123	149	−26
CRNM bleed	603	631	−28	603	714	−111
Treatment discontinuation due to adverse events	109	58	51	109	139	−30
Costs						
Anticoagulant costs	£1121	£98	£1023	£1121	£98	£1023
Monitoring and administration costs^a^	£63	£261	-£200	£63	£446	-£385
Event-related costs	£4069	£4214	-£145	£4069	£4170	-£101
QALYs	8.488	8.386	0.101	8.488	8.425	0.063
Life-years	10.421	10.311	0.110	10.421	10.375	0.046
Cost per QALY gained		£6692			£8528	

^a^Monitoring costs observed in the apixaban arm were attributed to the use of LMWH/VKA in patients who had a recurrent VTE event beyond the specified apixaban treatment period

Regardless of treatment duration for LMWH/VKA (i.e., initial only or initial plus extended), apixaban treatment yielded lower per-patient event costs (savings of £145 and £101, respectively) and lower per-patient monitoring and administration costs (savings of £200 and £385, respectively). However drug acquisition costs were higher for patients treated with apixaban in both analyses (incremental costs of £333 for initial only and £1023 for initial plus extended).

On average, a patient treated with apixaban accumulated 0.110 and 0.046 additional LYs as compared to initial treatment with LMWH/VKA and to initial plus extended treatment with LMWH/VKA, respectively. This translated to 0.101 and 0.063 additional QALYs compared to a patient treated with LMWH/VKA for initial or initial plus extended treatment, respectively.

These results led to an ICER of £6692 per QALY gained as compared to initial treatment with LMWH/VKA and an ICER of £8528 as compared to initial plus extended treatment with LMWH/VKA. The ICER’s indicate that initial plus extended treatment with apixaban is a cost-effective alternative to treatment with LMWH/VKA regardless of whether or not the latter is given for an extended period.

### One-way sensitivity analysis

**Fig. 2:**
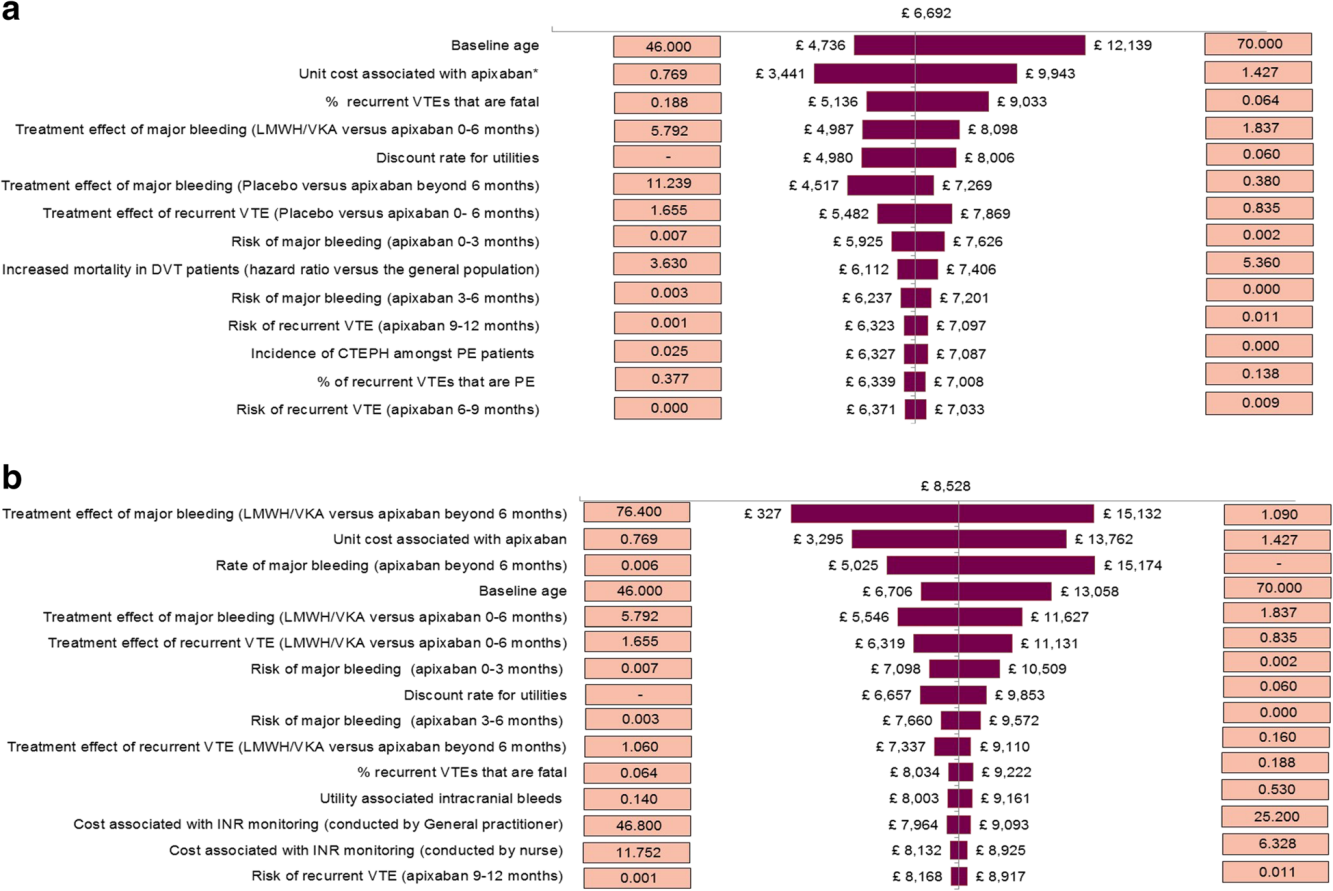
**a** One-way Sensitivity Analysis versus Initial LMWH/VKA. **b** One-way Sensitivity Analysis versus Initial plus Extended LMWH/VKA. The tornado diagrams (Fig. 2a and b) present the results of the deterministic sensitivity analyses and depict the key parameters that had the greatest impact on the ICER. The boxes to the right and left of the tornado diagram represent the range tested for the parameter detailed in the description. *The variation in the unit cost associated with apixaban corresponds to a range of the daily cost associated with apixaban of £3.07-£5.71 in the initial period and £1.54-£2.85 in the prolonged and extended period

Figure 2a and b present the results of the sensitivity analyses of the evaluation of initial plus extended treatment with apixaban versus initial or initial plus extended treatment with LMWH/VKA, respectively. These figures (known as ‘tornado diagrams’) show the 15 parameters that had the greatest effect on the ICERs arranged in descending order of such influence.

The results showed that incremental costs for apixaban versus initial and initial plus extended LMWH/VKA varied between £349 and £1008 and between £207 and £866, respectively, whilst incremental QALYs varied between 0.056 and 0.136 and 0.038 and 0.308, respectively. This resulted in ICERs varying between £3441 and £12,139 per QALY gained as compared to initial treatment with LMWH/VKA and between £327 and £15,132 per QALY gained as compared to initial plus extended treatment with LMWH/VKA.

When compared to initial treatment with LMWH/VKA (Fig. 2a), the most influential parameters were starting age, cost associated with apixaban, and the percentage of VTE-related deaths amongst recurrent VTEs. As compared to initial plus extended treatment with LMWH/VKA, the most influential parameters were relative risk of major bleeding versus apixaban for patients treated with VKA for an extended period, cost associated with apixaban, and the rate of major bleeding for patients treated with apixaban (Fig. 2b).

In both analyses, no scenario tested in the one-way sensitivity analysis resulted in an ICER above £20,000 per QALY gained. Therefore, the conclusion that apixaban is cost-effective is robust even when considering all plausible ranges of values of individual parameters.

### Probabilistic sensitivity analysis

**Fig. 3:**
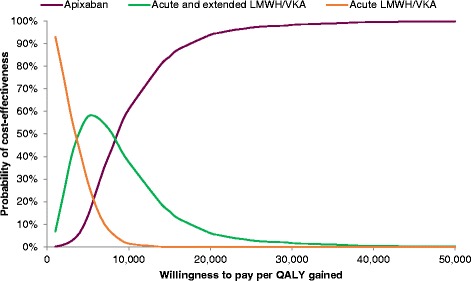
Cost-effectiveness Acceptability Curve. Figure 3 shows the probability of an intervention being the most cost-effective alternative at various willingness-to to-pay thresholds, given the uncertainty surrounding inputs

Results of the probabilistic analysis are depicted in terms of a cost-effectiveness acceptability curve, which shows the probability that a treatment is the most cost-effective alternative over a range of willingness-to-pay thresholds. At a willingness-to-pay threshold of £20,000 (denoting cost-effectiveness), initial plus extended treatment with apixaban had a 94% probability of being the most cost-effective treatment option over initial LMWH/VKA or initial plus extended LMWH/VKA. Based on these results, it appears that apixaban is a better treatment choice, economically speaking, over LMWH/VKA (initial, or initial plus extended) at any willing-to-pay threshold above about £8000 per QALY gained (Fig. 3).

### Scenario analysis of indefinite treatment

Evaluation of initial plus extended treatment with apixaban against alternative treatment durations demonstrated that the cost-effectiveness of apixaban is durable regardless of treatment duration for apixaban or LMWH/VKA. The cost associated with apixaban increases over indefinite treatment, as do health benefits. Although the base-case analysis versus initial plus extended LMWH/VKA suggests more VTE recurrences would occur in apixaban-treated patients, as a result of the favourable treatment effect of VKA in the extended period, this finding reversed when an indefinite treatment duration was assessed. Since apixaban’s safer bleeding profile allowed patients to remain on treatment for longer than they did VKA therapy, apixaban was predicted to prevent more recurrent VTE events over an indefinite duration.

## Discussion

This study assessed the cost-effectiveness in patients with VTE of initial plus extended treatment with apixaban versus LMWH/VKA provided either for initial treatment only, or for initial plus extended treatment. It demonstrated there are substantial benefits to extending treatment, with apixaban providing more health benefits that are cost-effective in comparison to extension with VKA.

Our analysis highlighted that initial plus extended treatment with apixaban was superior to treatment with LMWH/VKA in reducing the number of bleeding events over a patient’s lifetime, regardless of the duration for which LMWH/VKA was provided. The small net reductions in major bleeds and CRNM bleeds when compared to the initial treatment with LMWH/VKA reflects the fact that apixaban carries a lower risk of bleeding than does LMWH/VKA in the first 6 months of treatment, and that, compared with no treatment, it carries essentially no added risk of major bleeding (0.24% vs. 0.48% with no treatment) and only a small increase in the risk of CRNM bleeding (3.00% vs. 2.30%) [13]. The advantage with regards to bleeding effects was magnified when apixaban was compared to initial plus extended LMWH/VKA, in line with apixaban’s better bleeding profile than VKA when these two options have been compared indirectly for extended treatment [46]. Our analysis demonstrated an advantage for initial plus extended apixaban over initial treatment with 6 months of LMWH/VKA in preventing recurrent VTE events. However, when VKA treatment was used for an additional 12 months that is for a similar duration to apixaban, our analysis projected that it would be associated with slightly fewer VTE events than would apixaban. This effect was attributed to a slight advantage for VKA over apixaban in preventing recurrent VTE events in the extended period. This net increase in recurrent VTE events with apixaban was however offset by the reduction in bleeding events. Subsequently, the net reductions in clinical events with apixaban treatment as compared to either initial or initial plus extended LMWH/VKA were projected to result in an increased life expectancy and quality-adjusted life expectancy. These gains in health outcomes were accompanied by reductions in event-related costs and monitoring costs, but increased drug acquisition costs for apixaban which led to an overall increase in total costs. The ICER for initial plus extended treatment of apixaban versus initial LMWH/VKA was £6692 per QALY gained. When VKA was extended for 12 months post initial LMWH/VKA, the ICER was £8528 per QALY gained. The ICERs in both cases are below the commonly accepted threshold of £20,000 per QALY gained, [45] and so apixaban should be considered as a cost-effective alternative to LMWH/VKA in both scenarios.

In our analysis, we examined apixaban for 18 months and LMWH/VKA for 6 or 18 months, in line with treatment durations in clinical trials [12, 13]. However, the appropriate duration of anticoagulant treatment has been a source of clinical uncertainty. Guidelines on the duration of LMWH/VKA therapy suggest that patients should be treated for at least 3 months, and should be extended to at least 6 months in case of unprovoked VTE or indefinitely in patients with certain risk factors. In reality, the duration of treatment applied in clinical practice varies [4–7]. Therefore given the absence of an established duration of treatment for VTE patients, we used the clinical trial durations in our base case and conducted scenario analyses to determine the impact of using apixaban and LMWH/VKA over a lifetime duration. Compared with initial or initial plus extended lifelong treatment with LMWH/VKA, use of apixaban for the rest of a patient’s life (i.e., regarding VTE as a chronic condition) resulted in a cost-effectiveness estimate of £13,107 and £16,944 per QALY gained, respectively. Although the base base-case analysis comparison with initial plus extended LMWH/VKA predicted a higher number of VTE recurrences for apixaban, when treatment duration of both treatments was increased to lifetime, apixaban treatment was projected to lead to fewer VTE events than would lifetime LMWH/VKA.

Patients on apixaban experienced far fewer major bleeds than did those on VKA, which allowed them to stay on treatment longer. As a result, patients on apixaban for a lifelong duration experienced fewer recurrent VTEs, despite the better efficacy associated with VKA in the extended periods. The assumption that 52.7% of patients discontinued treatment after experiencing a major bleeding event was based on findings from AMPLIFY and is comparable to that adopted in earlier models (e.g. 40%, [15] 50% [47]) as observed in EINSTEIN. This conclusion held when one-third of patients or more were assumed to discontinue treatment after a major bleed. The improved safety of apixaban projected by the model coupled with findings from clinical trials, suggest that extended treatment can be clinically beneficial and cost-effective, thus, further challenging the current suggestion that there is clinical equipoise surrounding the use of extended anticoagulation.

Our model builds upon a foundation of structural similarities to earlier cost-effectiveness analyses that have also modelled VTE and bleeding events and the impact of anticoagulant treatment at several treatment durations [14, 15]. However, our projected QALYs are lower than those estimated with earlier models. This can be attributed to the fact that our model accounted for increased mortality associated with VTE, in addition to its explicit modelling of the excess mortality attributable to recurrent VTE and bleeding. This approach is consistent with evidence from retrospective studies showing excess mortality in patients who develop VTE, even after adjusting for the increase associated with recurrences and bleeding [32]. Also, our model accounted for increased risks of bleeding with aging [30]. This was in contrast to earlier models that assumed patients with VTE who do not experience any other events follow the mortality patterns of the general population, and which did not take into account the long-term impact of bleeding events [14, 15]. Had we utilised the same assumptions, the benefits associated with apixaban would have been higher due to longer life expectancy and increasing QALY gains to 0.146 and 0.081 compared with a 6-month and an 18-month treatment duration for LMWH/VKA, respectively, with corresponding ICERs of £6155 and £4134 per QALY gained. Our analysis utilised conservative inputs favouring comparators (for example, lower monitoring costs than those employed in earlier analyses), so the benefits of apixaban may be even greater than shown in our analysis [39]. In addition recent research has suggested that the true incidence of symptomatic CTEPH may be higher than the rate used in this analysis (4.4%; 95% CI 2.0–9.3 vs. 1.25%; 95% CI 0.03–2.46%, respectively) and therefore the reduction of CTEPH caused by apixaban anticoagulation may be even more pronounced [48]. Finally the recent reductions in the price associated with apixaban in the UK, would have resulted in even more favourable estimates of cost-effectiveness, further enhancing our conclusions that apixaban is a cost-effective alternative to LMWH/VKA.

Our study has a number of strengths. It is the first to examine the cost-effectiveness of apixaban for the treatment and prevention of VTE recurrence. In addition, we conducted scenario analyses to determine the impact of apixaban over varying treatment durations, thus, allowing clinicians to understand the overall health economic outcomes of the drug. Our analysis employs data from both initial-treatment and extended-treatment trials, [12, 13] thus, considering the whole of the available evidence base to project the potential impact of the introduction of apixaban. This serves as both a strength and limitation in our analysis for several reasons. In a controlled clinical trial setting, patients may receive improved care and exhibit enhanced adherence to the drug. Thus, the observed efficacy, safety, and tolerability may not reflect outcomes in the real world. In addition, AMPLIFY-EXT (which compared apixaban versus no treatment after initial LMWH/VKA was completed) reflects a patient population for whom the benefits of extended anticoagulation were uncertain (clinical equipoise), specifically excluding patients who were indicated for long-term anticoagulation. The populations studied in AMPLIFY and AMPLIFY-EXT therefore differ in terms of inclusion criteria, potentially excluding a proportion of the VTE population in the extended phase. However the trial characteristics were similar in other respects, and the approach of combining AMPLIFY and AMPLIFY-EXT data to determine apixaban’s cost-effectiveness over time has been accepted by the National Institute for Health and Care Excellence Appraisal Committee [49].

A further caveat to our analysis stems from the absence of head-to-head data for apixaban versus VKA in the extended period. Relative treatment effects were obtained by means of an NMA. However, this analysis did not control for the differences in patient baseline characteristics or key differences in the populations included in the trials. For example, out of the four VKA trials included in the analysed network, REMEDY [27] specifically included patients at high risk of VTE previously treated for 3–12 months, and LAFIT [4] and the WODIT [28, 29] trials included patients previously treated for 3 months, excluding those who had an indication for continuing oral anticoagulation therapy. AMPLIFY-EXT enrolled a population of patients previously treated for 6–12 months for whom there was clinical equipoise about the continuation of anticoagulation therapy [13]. Of note, shorter duration of previous anticoagulation is associated with increased risk of recurrent VTE [23]. So the differences in prior duration of treatment, with this being lower in the VKA trials, and the population inclusion criteria of the VKA trials, collectively suggest that the aggregation of VKA trials could result in the focus being on a higher-risk population. This could allow for the possibility of greater absolute risk reductions being suggested for VKA therapy in comparison to drugs such as apixaban that were evaluated in populations of patients at clinical equipoise with a longer prior treatment duration. We therefore believe that the limitations associated with the NMA make for conservative estimations of apixaban’s benefits, and we believe that the analyses we used represent the best available evidence in the absence of head-to-head trials. Moreover, the NMA used in this analysis is largely consistent with previously published NMAs [46].

Nonetheless the applicability of comparative data, derived from the NMA, to a real world setting is subject to additional uncertainty. Studies conducted in atrial fibrillation have suggested that a lower rate of major bleeding is observed in patients who are well controlled with VKA [50]. In addition emerging tools to assess which patients would likely have good time in therapeutic range with VKA treatment, could aid clinicians to identify which patients would do well on warfarin [51]. Reducing the relative risk of major bleeding for LMWH/VKA to the lowest estimate reported in real world studies [52, 53] (1.62 as compared to 3.33 and 7.70 in the first 6 months and beyond 6 months) did not alter the conclusions of our analysis. Apixaban remained cost-effective at thresholds below £30,000 per QALY gained.

Several other limitations are associated with our analysis, resulting from the modelling approach or other input data. The Markov cohort approach adopted is memory-less, in that, at any point in time, the risk of future events can only depend on the present state of patients, rather than the sequence of events that preceded it. Therefore due to the adopted approach, and for the sake of parsimony, we did not track or model any further VTE recurrences after the development of either IC bleeds or CTEPH. However, the expected cost and utility attempted to capture worse morbidity over the long-term and may implicitly account for future VTEs and bleeds. We also did not account for increased risk of additional recurrent VTEs once patients experienced a recurrent event. This assumption may be unfavourable to apixaban as compared to initial treatment with LMWH/VKA or favourable as compared to initial plus extended LMWH/VKA, given the reductions/increases observed in recurrent VTE events with apixaban treatment. However, sensitivity analysis that varied the costs and utilities of recurrent VTE events (which could be considered to reflect changes in the rates of recurrent VTEs) did not alter the conclusions of our evaluation.

Finally, several utility values were available for the health states included in the model. In our analysis, we attempted to use UK EQ-5D values where possible, but these were not available for all health states. Thus, utility values were obtained from several sources and may not be specific to a UK population. Of note, however, sensitivity analysis around the base-case utility values did not alter the conclusions of the evaluation. Similarly, results from one-way and probabilistic sensitivity analyses, in which all parameters that could be subject to uncertainty were varied over their confidence intervals, demonstrated that apixaban treatment was cost-effective regardless of the duration of treatment of with LMWH/VKA.

## Conclusions

The comprehensive assessment of the effects and costs of apixaban in this study predicted that initial plus extended treatment with apixaban for patients with VTE was cost-effective in the UK compared with LMWH/VKA used either for initial treatment only or initial plus extended treatment. Specifically, apixaban could offer favourable health benefits at a marginal increase in costs. The findings in our study could help clinicians and payers make informed decisions in the best interest of patients with VTE, particularly when considering extended anticoagulant treatment.

## Additional files

Additional file 1: The objective of this appendix is to provide additional technical documentation to the manuscript detailing the model calculations and data used to calculate the transition matrix in the model. (DOCX 26 kb)
Additional file 2: The following appendix details the results of the meta-analysis conducted to compare the clinical efficacy of apixaban and its relevant comparators for the treatment and secondary prevention of venous thromboembolism (VTE) in adult patients (≥18 years) who has received prior treatment for an acute VTE event. The meta-analysis was used to inform relative risk estimates in the economic model. (DOCX 149 kb)

## Abbreviations

AMPLIFY: Apixaban after the Initial Management of Pulmonary Embolism and Deep Vein Thrombosis with First-Line Therapy
AMPLIFY-EXT: AMPLIFY–Extended Treatment
BID: Twice a day
CRNM: Clinically relevant non-major
CTEPH: Chronic thromboembolic pulmonary hypertension
DVT: Deep vein thrombosis
IC: Intracranial
ICER: Incremental cost-effectiveness ratio
LAFIT: Long-term anticoagulation for a first episode of idiopathic venous thromboembolism
LMWH: Low-molecular-weight heparin
NHS: National Health Service
NMA: Network meta-analysis
PE: Pulmonary embolism
PTS: Post-thrombotic syndrome
QALY: Quality-adjusted life years
UK: United Kingdom
VKA: Vitamin K antagonists
VTE: Venous thromboembolism
WODIT: Warfarin Optimal Duration Italian Trial Investigators

## References

[1] Goldhaber SZ (2012). Venous thromboembolism: epidemiology and magnitude of the problem. Best Pract Res Clin Haematol.

[2] Cohen AT, Agnelli G, Anderson FA, Arcelus JI, Bergqvist D, Brecht JG (2007). Venous thromboembolism (VTE) in Europe. The number of VTE events and associated morbidity and mortality. Thromb Haemost.

[3] House of Commons Health Committee. The prevention of venous thromboembolism in hospitalised patients. Second report of session 2004–05. https://www.publications.parliament.uk/pa/cm200405/cmselect/cmhealth/99/99.pdf. Accessed 1 June 2014.

[4] Kearon C (1999). A comparison of 3 months of anticoagulation with extended anticoagulation for first episode of idiopathic venous thrmboembolism. N Engl J Med.

[5] Kearon C, Akl EA (2014). Duration of anticoagulant therapy for deep vein thrombosis and pulmonary embolism. Blood.

[6] Nicolaides AN, Fareed J, Kakkar AK, Comerota AJ, Goldhaber SZ, Hull R (2013). Prevention and treatment of venous thrmboembolism--international consensus statement. Int Angiol.

[7] Kaatz S, Qureshi W, Lavender RC (2011). Venous thromboembolism: what to do after anticoagulation is started. Cleve Clin J Med.

[8] Kearon C, Akl EA, Comerota AJ, Prandoni P, Bounameaux H, Goldhaber SZ (2012). Antithrombotic therapy for VTE disease: antithrombotic therapy and prevention of thrombosis, 9th ed: American College of Chest Physicians Evidence-based clinical practice guidelines. Chest.

[9] Le Gal G, Carrier M, Tierney S, Majeed H, Rodger M, Wells PS (2010). Prediction of the warfarin maintenance dose after completion of the 10 mg initiation nomogram: do we really need genotyping?. J Thromb Haemost.

[10] Gage BF, Cardinalli AB, Owens DK (1996). The effect of stroke and stroke prophylaxis with aspirin or warfarin on quality of life. Arch Intern Med.

[11] Keltai M, Keltai K (2011). New anticoagulants in the prevention and treatment of venous thromboembolism. Orv Hetil.

[12] Agnelli G, Buller HR, Cohen A, Curto M, Gallus AS, Johnson M (2013). Oral apixaban for the treatment of acute venous thromboembolism. N Engl J Med.

[13] Agnelli G, Buller HR, Cohen A, Curto M, Gallus AS, Johnson M (2013). Apixaban for extended treatment of venous thromboembolism. N Engl J Med.

[14] Seaman CD, Smith KJ, Ragni MV (2013). Cost-effectiveness of rivaroxaban versus warfarin anticoagulation for the prevention of recurrent venous thromboembolism: a U.S. perspective. Thromb Res.

[15] Lefebvre P, Coleman CI, Bookhart BK, Wang ST, Mody SH, Tran KN (2014). Cost-effectiveness of rivaroxaban compared with enoxaparin plus a vitamin K antagonist for the treatment of venous thromboembolism. J Med Econ.

[16] National Institute for Health and Care Excellence (NICE). Rivaroxaban for the treatment of deep vein thrombosis and prevention of recurrent deep vein thrombosis and pulmonary embolism. UK: NICE technology appraisals [TA261]; 2012.

[17] Sonnenberg FA, Beck JR (1993). Markov models in medical decision making: a practical guide. Med Decis Making.

[18] Siebert U, Alagoz O, Bayoumi AM, Jahn B, Owens DK, Cohen DJ (2012). State-transition modeling: a report of the ISPOR-SMDM modeling good research practices task force-3. Value Health.

[19] Briggs A, Sculpher M (1998). An introduction to Markov modelling for economic evaluation. Pharmacoeconomics.

[20] Lenert LA, Soetikno RM (1997). Automated computer interviews to elicit utilities: potential applications in the treatment of deep venous thrombosis. J Am Med Inform Assoc.

[21] Prandoni P (2012). Healthcare burden associated with the post-thrombotic syndrome and potential impact of the new oral anticoagulants. Eur J Haematol.

[22] National Institute for Health and Care Excellence (NICE). Final appraisal determination: Rivaroxaban for the treatment of deep vein thrombosis and prevention of deep vein thrombosis and pulmonary embolism, Issue date: May 2012, ID437. UK: NICE technology appraisals; 2012.

[23] Prandoni P, Noventa F, Ghirarduzzi A, Pengo V, Bernardi E, Pesavento R (2007). The risk of recurrent venous thromboembolism after discontinuing anticoagulation in patients with acute proximal deep vein thrombosis or pulmonary embolism. A prospective cohort study in 1626 patients. Haematologica.

[24] Miniati M, Monti S, Bottai M, Scoscia E, Bauleo C, Tonelli L (2006). Survival and restoration of pulmonary perfusion in a long-term follow-up of patients after acute pulmonary embolism. Medicine (Baltimore).

[25] Prandoni P, Villalta S, Bagatella P, Rossi L, Marchiori A, Piccioli A (1997). The clinical course of deep-vein thrombosis. Prospective long-term follow-up of 528 symptomatic patients. Haematologica.

[26] Kahn SR, Shapiro S, Wells PS, Rodger MA, Kovacs MJ, Anderson DR (2014). Compression stockings to prevent post-thrombotic syndrome: a randomised placebo-controlled trial. Lancet.

[27] Schulman S, Kearon C, Kakkar AK, Schellong S, Eriksson H, Baanstra D (2013). Extended use of dabigatran, warfarin, or placebo in venous thromboembolism. N Engl J Med.

[28] Agnelli G, Prandoni P, Santamaria MG, Bagatella P, Iorio A, Bazzan M (2001). Three months versus 1 year of oral anticoagulant therapy for idiopathic deep venous thrombosis. Warfarin optimal duration Italian trial investigators. N Engl J Med.

[29] Agnelli G, Prandoni P, Becattini C, Silingardi M, Taliani MR, Miccio M (2003). Extended oral anticoagulant therapy after a first episode of pulmonary embolism. Ann Intern Med.

[30] Ariesen M, Claus S, Rinkel G, Algra A (2003). Risk factors for intracerebral hemorrhage in the general population: a systematic review. Stroke.

[31] Human Mortality Database. 2011 UK life tables. http://www.mortality.org/. Accessed 1 Oct 2013.

[32] Flinterman LE, van Hylckama VA, Cannegieter SC, Rosendaal FR (2012). Long-term survival in a large cohort of patients with venous thrombosis: incidence and predictors. Plos Med.

[33] Ng AC, Chung T, Yong AS, Wong HS, Chow V, Celermajer DS (2011). Long-term cardiovascular and noncardiovascular mortality of 1023 patients with confirmed acute pulmonary embolism. Circ Cardiovasc Qual Outcomes.

[34] Prandoni P, Trujillo-Santos J, Sanchez-Cantalejo E, Dalla Valle F, Piovella C, Pesavento R (2010). Major bleeding as a predictor of mortality in patients with venous thromboembolism: findings from the RIETE registry. J Thromb Haemost.

[35] Curtis L (2012). Unit costs of health and social care 2012. Personal social services research unit.

[36] British National Formulary (BNF). http://www.medicinescomplete.com/mc/bnf/current/index.htm. Accessed 1 Oct 2013.

[37] Commercial Medicines Unit (CMU). Electronic Market Information Tool (eMIT). https://www.gov.uk/government/publications/drugs-and-pharmaceutical-electronic-market-information-emit. Accessed 1 Oct 2013.

[38] National Schedule of Reference Costs Year: 2011–12. All NHS trusts and NHS foundation trusts-HRG Data.

[39] Copley V, Pickett K, Cooper K, et al. Rivaroxaban for the treatment of pulmonary embolism and the prevention of recurrent venous thromboembolism. A single technology appraisal. In.; 2013.

[40] Kind P, Dolan P, Gudex C, Williams A (1998). Variations in population health status: results from a United Kingdom national questionnaire survey. BMJ.

[41] Locadia M, Bossuyt PM, Stalmeier PF, Sprangers MA, van Dongen CJ, Middeldorp S (2004). Treatment of venous thromboembolism with vitamin K antagonists: patients’ health state valuations and treatment preferences. Thromb Haemost.

[42] Sullivan PW, Slejko JF, Sculpher MJ, Ghushchyan V (2011). Catalogue of EQ-5D scores for the United Kingdom. Med Decis Making.

[43] National Institute for Health and Care Excellence (NICE). Venous thromboembolism: reducing the risk: Reducing the risk of venous thromboembolism (deep vein thrombosis and pulmonary embolism) in patients admitted to hosptial. NICE guidelines [CG92}. UK: NICE technology appraisals; 2010.

[44] Hogg K, Kimpton M, Carrier M, Coyle D, Forgie M, Wells P (2013). Estimating quality of life in acute venous thrombosis. JAMA Intern Med.

[45] National Institute for Health and Care Excellence (NICE). Guide to the methods of technology appraisal 2013. https://www.nice.org.uk/process/pmg9/chapter/foreword. Accessed 1 Oct 2013. 27905712

[46] Castellucci LA, Cameron C, Le Gal G, Rodger MA, Coyle D, Wells PS (2013). Efficacy and safety outcomes of oral anticoagulants and antiplatelet drugs in the secondary prevention of venous thromboembolism: systematic review and network meta-analysis. BMJ..

[47] National Institute for Health and Care Excellence (NICE). Venous thromboembolism (treatment and long term secondary prevention) - rivaroxaban: manufacturer submission. UK: NICE technology appraisals; 2012.

[48] Dentali F, Donadini M, Gianni M, Bertolini A, Squizzato A, Venco A (2009). Incidence of chronic pulmonary hypertension in patients with previous pulmonary embolism. Thromb Res.

[49] National Institute for Health and Care Excellence (NICE). Final appraisal determination document: Apixaban for the treatment and secondary prevention of deep vein thrombosis and/or pulmonary embolism. UK: NICE technology appraisals; 2015.

[50] Ruff CT, Giugliano RP, Braunwald E, Hoffman EB, Deenadayalu N, Ezekowitz MD (2014). Comparison of the efficacy and safety of new oral anticoagulants with warfarin in patients with atrial fibrillation: a meta-analysis of randomised trials. Lancet.

[51] Apostolakis S, Sullivan RM, Olshansky B, Lip GY (2013). Factors affecting quality of anticoagulation control among patients with atrial fibrillation on warfarin: the SAMe-TT(2)R(2) score. Chest.

[52] Lip G, Keshishian A, Kamble S, Pan X, Burns L, Mardekian J (2016). Real world comparison of major bleeding risk among Non-valvular atrial fibrillation patients newly initiated on apixaban, warfarin, dabigatran or rivaroxaban: a 1:1 propensity-score matched analysis. J Am Coll Cardiol.

[53] Lamberts M, Harboe L, Lefevre C, Evans D, Gislason G (2016). Comparison of bleeding and treatment persistence among new users of novel oral anticoagulants and warfarin in patients with non-valvular atrial fibrillation. J Am Coll Cardiol.

[54] Luengo-Fernandez R, Gray AM, Rothwell PM (2012). Oxford vascular study. A population-based study of hospital care costs during 5 years after transient ischemic attack and stroke. Stroke.

[55] Ghofrani HA, D’Armini AM, Grimminger F, Hoeper MM, Jansa P, Kim NH (2013). Riociguat for the treatment of chronic thromboembolic pulmonary hypertension. N Engl J Med.

