# Supplementary material for: Cost-effectiveness of apixaban versus low molecular weight heparin/vitamin k antagonist for the treatment of venous thromboembolism and the prevention of recurrences
Source: BMC Health Serv Res. 2017 Jan 23;17:74. doi: 10.1186/s12913-017-1995-8 (PMC5259920; doi:10.1186/s12913-017-1995-8)
Supplement: Additional file 1: — The objective of this appendix is to provide additional technical documentation to the manuscript detailing the model calculations and data used to calculate the transition matrix in the model. (DOCX 26 kb) [file 12913_2017_1995_MOESM1_ESM.docx]

**Supplementary Appendix**

Supplementary Appendix A to Lanitis et al. “Cost-effectiveness of apixaban versus low molecular weight heparin/vitamin k antagonist for the treatment of venous thromboembolism and the prevention of recurrences”

# objective

The objective of this appendix is to provide additional technical documentation to the manuscript entitled “Cost-effectiveness of apixaban versus low molecular weight heparin/vitamin k antagonist for the treatment of venous thromboembolism and the prevention of recurrences”. In the sections below we provide further details on the model calculations.

# MODEL APPROACH

As described in the manuscript, all patients in the cohort were assumed to begin in the index venous thromboembolism (VTE) health state on treatment, having just experienced a VTE event and commenced anticoagulant treatment. The index VTE health state on treatment is segregated into patients with index pulmonary embolism (PE) and patients with index deep vein thrombosis (DVT), to account for differing risks for chronic thromboembolic pulmonary hypertension (CTEPH) (only in index PE patients or patients who experience recurrent PE [within index DVT]) and post thrombotic syndrome (PTS) (only in index DVT patients or patients who experience recurrent DVT [within index PE]). During each cycle, the cohort was subjected to competing risks of the following events: recurrent VTE, major bleeds, clinically relevant non-major (CRNM) bleeds, CTEPH and death. Recurrent VTE events were classified as non-fatal recurrent PE, non-fatal recurrent DVT, or VTE-related death. Major bleeding events were classified as fatal and or non-fatal; and those that were non-fatal were further segregated between intracranial (IC) bleeds and non-IC bleeds. Patients were also at risk of treatment discontinuation, either as a result of major bleeding or because of adverse events unrelated to bleeding, in which case patients would move to ‘VTE off-treatment’ health states, where they would be exposed to the same events but at higher risk levels, in line with the fact that they were not receiving anticoagulant treatment. The proportion of patients with PTS was also estimated in each cycle, however was not considered a separate health state. The model steps through multiple cycles until all patients from the initial cohort are dead.

To determine the transition risks in each cycle, 3 monthly risks were obtained from the AMPLIFY and AMPLIFY-EXT trials as detailed in Table 1 of the manuscript. Where annual event rates were obtained these were converted to three monthly risks using the following formula (where p=probability, r=rate, t=time):

$$p=1-e^{-rt}$$

To determine the risks for patients treated with LMWH/VKA the relative risks displayed in Table 2 in the manuscript were multiplied by the risks of events for patients treated with apixaban as displayed in Table 1 of the manuscript.

The transition risks from the index PE on treatment health state in the first cycle are displayed in Table A1. As noted in the manuscript 34.2% of patients were assumed to have index PE. Transition risks are only displayed for the first cycle, thus subsequent transitions from each health state are not displayed, but can be calculated in the same manner using the cohort distribution in previous cycles and the time dependent risks detailed in the manuscript.

Table A1 Transition Matrix from Index PE state in first cycle

|  | **Apixaban** | **LMWH/VKA** | **Note** |
| --- | --- | --- | --- |
| **Index PE** | **=0.3152 + 0.0022+0.0024 + 0.0002+0.0010+0.0090+ 0.0005+0.0082+0.0033=0.3420** | **= 0.2972 + 0.0027 + 0.0030 + 0.0006 + 0.0036 + 0.0216 + 0.0005 + 0.0088 + 0.0041 = 0.3420** |  |
| **Recurrent VTE** |  |  |  |
| *VTE related death & recurrent VTE* | *=0.342*0.0171= 0.0058* | *=0.342*0.0205=0.0070* |  |
| VTE related death | = 0.0058*22% = 0.0013 | = 0.0070*22%= 0.0015 | Included in death state |
| Recurrent PE | = 0.0058*38% = 0.0022 | = 0.0070*38% = 0.0026 | Included in index PE health state at the beginning of the next cycle to be subjected to the same risks in subsequent cycles |
| Recurrent DVT | = 0.0058*41% = 0.0024 | = 0.0070*41% = 0.0028 |  |
| **Bleeding** |  |  |  |
| *Major bleeds* | *=0.342*0.0041=0.0014* | *=0.342*0.00137=0.0047* |  |
| Incident fatal major bleeds | = 0.0014*13% = 0.0002 | = 0.0047*13% = 0.00063 | Included in death state |
| Incident nonfatal major bleed | = 0.0014-0.0002 = 0.0012 | = 0.0047-0.000623 = 0.0040 |  |
| Incident nonfatal IC bleed | = 0.0012*14% = 0.0002 | = 0.0040*14% = 0.00056 | Included in post IC bleed health state at the beginning of the next cycle |
| Incident nonfatal non-IC bleed | = 0.0012-0.0002 = 0.0010 | = 0.0042-0.00056 = 0.0035 | Included in index PE health state at the beginning of the next cycle to be subjected to the same risks in subsequent cycles |
| Post IC bleed - cumulative | = (0+0)*(1-0.0140) = 0 | = (0+0)*(1-0.0140) = 0 | In this calculation the first 0 represents state membership in the previous cycle and the second 0 represents incident IC bleeds in the previous cycle. In subsequent cycles incident cases are added to the cumulative total and death is subtracted. |
| Death from post IC bleed state | = (0+0)*0.0140 = 0 | = (0+0)*0.0140 = 0 | Included in death state |
| *CRNM bleed* | = 0.342*0.0265 = 0.0091 | = 0.342*0.0552 = 0.0189 | Included in index PE health state at the beginning of the next cycle to be subjected to the same risks in subsequent cycles |
| **Chronic thromboembolic pulmonary hypertension** |  |  |  |
| *CTEPH* | *= 0.3420*0.0015= 0.0005* | *= 0.3420*0.0015= 0.0005* |  |
| Post CTEPH-cumulative | = (0+0)*(1-0.0070) = 0 | = (0+0)*(1-0.0070) = 0 | In this calculation the first 0 represents state membership in the previous cycle and the second 0 represents incident CTEPH in the previous cycle. In subsequent cycles incident cases are added to the cumulative total and death is subtracted. |
| Death from post CTEPH state | = (0+0)*0.0070= 0 | = (0+0)*0.0070= 0 | Included in death state |
| **Treatment discontinuation** |  |  |  |
| Index PE off treatment | = 0.3420*0.0240+(0*53%) = 0.0082 | = 0.3420*0.0257+(0*53%) = 0.0088 | All patients in the index PE on treatment health state are subjected to risks of treatment discontinuation. In addition patients experiencing non-fatal non IC bleeds have a risk of discontinuing treatment (53%). |
| **Death** | 0.0033 | 0.0040 |  |
| Event related death | = 0.0013 + 0.0002 + 0 + 0 =0.0014 | = 0.0015 + 0.00063 + 0 + 0 = 0.0021 | Deaths due to bleeds, VTE related death and CTEPH |
| Background incident death† | = 0.3420 * 0.0054 = 0.0019 | = 0.3420 * 0.0054 = 0.0019 |  |

Note: Risks of recurrent VTE, major bleeds, CRNM bleeds, and CTEPH are applied to the cohort remaining alive thus the risk of transition to each event is adjusted e.g. the risk of *VTE related death & recurrent VTE* for apixaban is calculated as 1*(1-0.0054)*0.0171, however the calculations presented here have excluded this to demonstrate how the clinical data has been translated.

† Risk of death varies each cycle and by gender
