# Supplementary material for: Cost-effectiveness of apixaban versus low molecular weight heparin/vitamin k antagonist for the treatment of venous thromboembolism and the prevention of recurrences
Source: BMC Health Serv Res. 2017 Jan 23;17:74. doi: 10.1186/s12913-017-1995-8 (PMC5259920; doi:10.1186/s12913-017-1995-8)
Supplement: Additional file 2: — The following appendix details the results of the meta-analysis conducted to compare the clinical efficacy of apixaban and its relevant comparators for the treatment and secondary prevention of venous thromboembolism (VTE) in adult patients (≥18 years) who has received prior treatment for an acute VTE event. The meta-analysis was used to inform relative risk estimates in the economic model. (DOCX 149 kb) [file 12913_2017_1995_MOESM2_ESM.docx]

**Supplementary Appendix to Lanitis et al. “Cost-effectiveness of anticoagulation with apixaban versus low molecular weight heparin/vitamin k antagonist for the treatment of venous thromboembolism and the prevention of recurrences”**

Objective

The following report details the results of the meta-analysis conducted to compare the clinical efficacy of apixaban and its relevant comparators for the treatment and secondary prevention of venous thromboembolism (VTE) in adult patients (≥18 years) who has received prior treatment for an acute VTE event. The meta-analysis was used to inform relative risk estimates in the economic model. **The methods and data presented here are under review for publication.**

Methodology

Scope

The meta-analysis was conducted based on prospective Phase III randomized controlled trials (RCTs) with either open-label, or double-blind study designs comparing new oral anticoagulants (NOACs) with the current standard of care for VTE (no further treatment). A systematic review was conducted to identify potentially relevant studies for inclusion in the meta-analysis. The scope of the current meta-analysis detailed in this report is restricted to those patients receiving extended anticoagulation for a VTE event with one of the interventions listed below. The following additional inclusion criteria were applied to identify RCTs reporting outcome data for this population.

Population:

- Patients who had received prior treatment for an acute VTE event

Interventions of interest:

- NOACs:
  - Apixaban
  - Dabigatran
  - Rivaroxaban
- Aspirin
- Warfarin (standard dose – INR 2.0-3.0)
- Warfarin (low dose – INR 1.5-1.9)

Comparators:

- Placebo
- Warfarin (standard dose)

Outcomes

Efficacy:

- Recurrent VTE and VTE-related death
- Overall treatment discontinuation

Safety:

- Major bleeding
- CRNM bleeding

Studies Included in the Meta-analysis

Eleven RCTs (reported in ten publications) reporting on seven treatments were included in the analysis of extended treatment:

- AMPLIFY-EXT [[1](#_ENREF_1)] – apixaban 2.5mg BD or apixaban 5mg BD vs. placebo
- EINSTEIN-EXT [[2](#_ENREF_2)] – rivaroxaban 20mg OD vs. placebo
- WARFASA [[3](#_ENREF_3)] – aspirin 100mg OD vs. placebo
- RESONATE [[4](#_ENREF_4)] – dabigatran 150mg BD vs. placebo
- REMEDY [[4](#_ENREF_4)] – dabigatran 150mg BD vs. warfarin INR 2.0-3.0
- ASPIRE [[5](#_ENREF_5)] – aspirin 100mg OD vs. placebo
- LAFIT [[6](#_ENREF_6)] – warfarin INR 2.0-3.0 vs. placebo
- ELATE [[7](#_ENREF_7)] – warfarin INR 1.5-1.9 vs. warfarin 2.0-3.0
- WODIT DVT [[8](#_ENREF_8)] – VKA continuation vs. VKA discontinuation / observation
- WODIT PE [[9](#_ENREF_9)] VKA continuation vs. VKA discontinuation / observation
- PREVENT [[10](#_ENREF_10)] – warfarin INR 1.5-1.9 vs. placebo

Comparisons between all treatments were made based on the network shown in Figure 1.

Figure 1. Extended network

**Statistical Analysis**

*Data Assumptions*

Event data were captured so as to include all events occurring while patients were followed during the planned intervention period, as defined in the pre-specified study protocol. This was consistent with the approach adopted by Castellucci et al [[11](#_ENREF_11)].

The primary analyses used the outcome event data reported while patients were on treatment, with the exception of the ASPIRE [[5](#_ENREF_5)] and ELATE [[7](#_ENREF_7)] trials, where the outcome data were taken as that reported while the patients were in the follow-up period of the study, having stopped treatment because this was the specified analysis plan of these trials.

*Zero Events*

In order to calculate the study level relative risks using a frequentist approach an arbitrary constant was added to cells in order to obtain non-infinite estimates of treatment effects and non-infinite variance [[12](#_ENREF_12)]. That is, in the case where a study contained a zero observation (e.g. no patients had the event), by default, a continuity correction was applied where 0.5 was added to each cell of the study outcome results table within that study in order to calculate the study level relative risk [[12](#_ENREF_12), [13](#_ENREF_13)].

A major strength of the Bayesian Markov Chain Monte Carlo (MCMC) approach is that zero cells are allowed and special precautions are not usually needed in the case of the occasional trial with a zero cell count [[12](#_ENREF_12)]. In extreme cases such as those where several trials have zero events or many of the trials are small the models may be numerically unstable either failing to converge, or converging to a posterior with very high standard deviation [[12](#_ENREF_12)]. A specific problem arises in sparse networks where for example there is one trial per treatment comparison [[12](#_ENREF_12)]. If this trial contains a zero event then it may not be possible to estimate a treatment effect. A solution to this issue is to revert to the practice of assigning a continuity correction (as per the frequentist approach). In the instances where there were zero events in treatment arms, models were run without a continuity correction. If the model failed or did not converge then a continuity correction was applied.

*Network Meta-analysis Methodology*

The analysis was conducted using NMA. The main motivation for carrying out an NMA is the ability to synthesize all available evidence on an entire network of treatments and estimate the relative efficacy of each treatment compared with all comparators within a single analysis.

NMA is based on direct and indirect comparisons of differences between randomized treatment allocations and therefore overcomes the biases inherent within “naïve” comparisons of outcomes in individual arms. By considering all of the evidence, NMA can therefore give more precise estimates of treatment effect and reflect the full range of estimates from the literature, taking account of inconsistencies and heterogeneity. It is a practice endorsed by the International Society for Pharmacoeconomics and Outcomes Research (ISPOR) for the purpose of using indirect and direct evidence for decision making. [[14](#_ENREF_14)].

The NMA was conducted under a Bayesian framework using the statistical software package WinBUGS. In fixed-effects meta analyses, we assume that treatment effects can be estimated directly from the trial data, while in random-effects meta-analysis we assume that the treatment effects are drawn from a common distribution with a variance parameter equal to the between-studies variance, or heterogeneity. A random-effects model is more complex than a fixed-effects model as it requires more parameters, and therefore the added flexibility means it will usually provide a better fit to the data. However, as with all statistical modelling, it is important to find a trade-off between improved fit and added complexity in order to make meaningful inferences. Therefore a more complex model should only be preferred if it provides an improvement in model fit substantial enough to justify its added complexity.

The model fit of the fixed- and random-effects models conducted for each outcome was compared using the deviance information criterion (DIC). In comparing models, the fixed-effects model was considered to be the model of choice unless the DIC of the random-effects model was at least 3 -5 points lower than that of the fixed-effects model, thereby providing sufficient improvement in model fit to justify the additional complexity of a random-effects model [[15](#_ENREF_15)]. For all the outcomes, the random-effects models were deemed to be no better fit than the fixed-effects based on the DIC (differences between values all <3). Model fit was also assessed by comparing the residual deviance of the model with the number of data points in the model. Models were judged to be of reasonable fit where the average residual deviance (i.e. residual deviance divided by number of data points) was close to 1. The between-study precision was examined for each random-effects model, but all analyses proved to be underpowered to estimate the between study heterogeneity. Therefore, the fixed-effect models were considered to be the most robust models and these data are presented in the current report.

Results of Network Meta-analysis

VTE and VTE-related Death

Table 1. Data used in analysis of VTE and VTE-related death

| **Study** | **Treatment arm** | **No. of patients (N)** | **Number of patients with an event (n)** | **Relative risk**  **(95% CI)** |
| --- | --- | --- | --- | --- |
| AMPLIFY-EXT [[1](#_ENREF_1)] | Apixaban 2.5 mg BD | 840**^†^** | 14^‡^ | **0.19^#^**  **(0.11, 0.33)** |
|  | Apixaban 5 mg BD | 813**^†^** | 14^‡^ | **0.20^#^**  **(0.11, 0.34)** |
|  | Placebo | 829**^†^** | 73^‡^ | **-** |
| EINSTEIN-EXT [[2](#_ENREF_2)] | Rivaroxaban 20 mg OD | 602^§^ | 8^¶^ | **0.19**  **(0.09, 0.40)** |
|  | Placebo | 594^§^ | 42^¶^ |  |
| WARFASA [[3](#_ENREF_3)] | Aspirin 100 mg OD | 205^††^ | 23^‡‡^ | **0.57**  **(0.35, 0.91)** |
|  | Placebo | 197^††^ | 39^‡‡^ |  |
| RESONATE [[4](#_ENREF_4)] | Dabigatran 150 mg BD | 681^§§^ | 3^†††^ | **0.08**  **(0.02, 0.25)** |
|  | Placebo | 662^§§^ | 37^†††^ |  |
| REMEDY [[4](#_ENREF_4)] | Dabigatran 150 mg BD | 1430^§§^ | 26^¶¶^ | 1.44  (0.79, 2.62) |
|  | Warfarin INR 2.0-3.0 | 1426^§§^ | 18^¶¶^ |  |
| ASPIRE [[5](#_ENREF_5)] | Aspirin 100 mg OD | 411^‡‡‡^ | 57^§§§^ | 0.78  (0.57, 1.07) |
|  | Placebo | 411^‡‡‡^ | 73^§§§^ |  |
| LAFIT [[6](#_ENREF_6)] | Warfarin INR 2.0-3.0 | 79^‡‡‡^ | 1^¶¶¶¶^ | **0.06**  **(0.01, 0.45)** |
|  | Placebo | 83^‡‡‡^ | 17^¶¶¶¶^ |  |
| ELATE [[7](#_ENREF_7)] | Warfarin INR 1.5-1.9 | 369^‡‡‡^ | 16^¶¶¶^ | **2.67**  **(1.06, 6.74)** |
|  | Warfarin INR 2.0-3.0 | 369^‡‡‡^ | 6^¶¶¶^ |  |
| WODIT DVT [[8](#_ENREF_8)] | VKA continuation | 134^†^ | 1^‡‡‡‡^ | **0.09**  **(0.012, 0.69)** |
|  | VKA discontinuation / observation | 133^†^ | 11^††††^ |  |
| PREVENT [[10](#_ENREF_10)] | Warfarin INR 1.5-1.9 | 255^‡‡‡^ | 14^§§§§^ | **0.38**  **(0.21, 0.68)** |
|  | Placebo | 253^‡‡‡^ | 37^§§§§^ |  |

BD, twice daily; CI, confidence interval; INR, international normalized ratio; OD, once daily

Significant results are in bold.

# Relative risk represents treatment versus placebo.

†Patients included in ITT analysis

‡Reported as ‘recurrent VTE or VTE-related death’ as reported in Table 2 of the publication [[1](#_ENREF_1)]

§Number of patients included in the ITT analysis

¶Reported as ‘recurrent VTE’ in Table 4 of publication [[2](#_ENREF_2)]

††Modified ITT population: all patients who received at least one dose of the assigned study drug post randomisation.

‡‡Reported as recurrent VTE episodes during treatment in Table 2 of publication [[3](#_ENREF_3)]

§§Modified ITT: excluding patients who did not receive any dose of the study drug

¶¶Reported as ‘recurrent or fatal VTE’ in Table 2 of publication [[4](#_ENREF_4)]

†††Reported as ‘recurrent or fatal VTE or unexplained death’ in Table 3 of publication [[4](#_ENREF_4)]

‡‡‡All randomised patients

§§§Reported as recurrent VTE (composite of symptomatic, objectively confirmed DVT, non-fatal PE, or fatal PE) in Table 2 of the publication [[5](#_ENREF_5)]

¶¶¶Reported as recurrent VTE on p.636 of the publication [[7](#_ENREF_7)]

††††Reported as risk of VTE recurrence during the first 9 months of follow up on p.168 of publication [[8](#_ENREF_8)]

‡‡‡‡Reported as risk of VTE recurrence during the first 9 months of follow up on p.168 of publication [[8](#_ENREF_8)]. Although 4 patients reported VTE recurrence during the first 9 months of follow up, 3 patients had prematurely interrupted oral anticoagulant therapy and therefore only 1 patient had a recurrence while receiving active oral anticoagulant therapy.

§§§§Reported as ‘recurrent VTE’ in Table 2 of the publication [[10](#_ENREF_10)].

¶¶¶¶Reported as ‘VTE’ in Table 2 of the publication [[6](#_ENREF_6)]

Table 2. Relative risk fixed effect NMA results for VTE and VTE-related death (treatment vs. comparator)

| **Treatment** | **Comparator** | **Relative risk (95% Crl)**  **(median)** |
| --- | --- | --- |
| Warfarin INR 2.0-3.0 | Apixaban 2.5 mg BD | 0.42 (0.16, 1.06) |

BD, twice daily; CrI, credible interval; INR, international normalized ratio; OD, once daily

Major Bleeding

Table 3. Data used in analysis of major bleeding

| **Study** | **Treatment arm** | **No. of patients (N)** | **Number of patients with an event (n)** | **Relative risk**  **(95% CI)** |
| --- | --- | --- | --- | --- |
| AMPLIFY-EXT [[1](#_ENREF_1)] | Apixaban 2.5 mg BD | 840^†^ | 2^‡^ | 0.49^#^  (0.09, 2.68) |
|  | Apixaban 5 mg BD | 811^†^ | 1^‡^ | 0.26^#^  (0.03, 2.27) |
|  | Placebo | 826^†^ | 4^‡^ |  |
| EINSTEIN-EXT [[2](#_ENREF_2)] | Rivaroxaban 20 mg OD | 598^§^ | 4^¶^ | 8.88  (0.48, 164.57) |
|  | Placebo | 590^§^ | 0^¶,*^ |  |
| WARFASA [[3](#_ENREF_3)] | Aspirin 100 mg OD | 205^††^ | 1^‡‡^ | 0.96  (0.06, 15.26) |
|  | Placebo | 197^††^ | 1^‡‡^ |  |
| RE-SONATE [[4](#_ENREF_4)] | Dabigatran 150 mg BD | 684^¶¶^ | 2^†††^ | 4.82  (0.23, 100.16) |
|  | Placebo | 659^¶¶^ | 0^†††,*^ |  |
| RE-MEDY [[4](#_ENREF_4)] | Dabigatran 150 mg BD | 1430^§§^ | 13^†††^ | 0.52  (0.27, 1.01) |
|  | Warfarin INR 2.0-3.0 | 1426^§§^ | 25^†††^ |  |
| ASPIRE [[5](#_ENREF_5)] | Aspirin 100 mg OD | 411^‡‡‡^ | 8^§§§^ | 1.33  (0.47, 3.81) |
|  | Placebo | 411^‡‡‡^ | 6^§§§^ |  |
| LAFIT [[6](#_ENREF_6)] | Warfarin INR 2.0-3.0 | 79^‡‡‡^ | 3^§§§§^ | 7.35  (0.39, 140.09) |
|  | Placebo | 83^‡‡‡^ | 0^§§§§,*^ |  |
| ELATE [[7](#_ENREF_7)] | Warfarin INR 1.5-1.9 | 369^‡‡‡^ | 9^¶¶¶^ | 1.13  (0.44, 2.88) |
|  | Warfarin INR 2.0-3.0 | 369^‡‡‡^ | 8^¶¶¶^ |  |
| WODIT DVT [[8](#_ENREF_8)] | VKA continuation | 134^‡‡‡^ | 4^††††^ | 1.99  (0.37, 10.66) |
|  | VKA discontinuation / observation | 133^‡‡‡^ | 2^††††^ |  |
| PREVENT [[10](#_ENREF_10)] | Warfarin INR 1.5-1.9 | 255^‡‡‡^ | 5^‡‡‡‡^ | 2.48  (0.49, 12.67) |
|  | Placebo | 253^‡‡‡^ | 2^‡‡‡‡^ |  |
| WODIT PE [[9](#_ENREF_9)] | VKA continuation | 165^‡‡‡^ | 3^¶¶¶¶^ | 2.93  (0.31, 27.85) |
|  | VKA discontinuation / observation | 161^‡‡‡^ | 1^¶¶¶¶^ |  |

BD, twice daily; CI, confidence interval; INR, international normalized ratio; OD, once daily

# Relative risk represents treatment versus placebo.

* There were insufficient patient / trial data to ensure the WinBUGS model converged without applying a continuity correction. Therefore, where zero outcome events were reported, a correction of 0.5 was added as per the Cochrane Handbook.

†Patients included in safety analysis: data from patients during the time they were receiving treatment, defined as the time between administration of the first dose of a study drug and 48 hours after administration of the last dose.

‡Reported as ‘major bleeding’ as reported in Table 2 of the publication [[1](#_ENREF_1)]

§Safety analyses included all patients who received the assigned study drug. Bleeding events were included in the analysis if they occurred during treatment or within 2 days after discontinuation of the study drug.

¶Reported as ‘major bleeding’ in Table 4 of publication [[2](#_ENREF_2)] [some patients had more than 1 event].

††All patients who received at least one dose of the study drug.

‡‡Reported as ‘major bleeding’ in Table 2 of publication [[3](#_ENREF_3)]

§§The safety analysis was based on the treatment period and a 3-day washout period after the end of treatment.

¶¶The safety analysis included 684 patients who received dabigatran and 659 who received placebo. Three patients in the placebo group mistakenly received dabigatran throughout the study.

†††Reported as ‘major bleeding events’ in Table 2 (RE-MEDY) and Table 3 (RE-SONATE) of publication [[4](#_ENREF_4)]

‡‡‡All randomised patients

§§§Reported as ‘major bleeding’ in Table 2 of the publication [[5](#_ENREF_5)]

¶¶¶Reported as ‘major bleeding episode’ in Table 2 of the publication [[7](#_ENREF_7)]

††††Reported as ‘major bleeding’ in Table 2 of publication [[8](#_ENREF_8)]

‡‡‡‡Reported as ‘major bleeding episode’ in Table 2 of the publication [[10](#_ENREF_10)].

§§§§Reported as ‘Type of bleeding: major’ in Table 2 of the publication [[6](#_ENREF_6)]

¶¶¶¶Reported as major bleeding in Table 3 of the publication [[9](#_ENREF_9)]. Among patients randomised to continue therapy, all bleeding episodes occurred during treatment.

Table 4. Relative risk fixed effects NMA results for major bleeding (treatment vs. comparator)

| **Treatment** | **Comparator** | **Relative risk (95% CrI) (median)** |
| --- | --- | --- |
| Warfarin INR 2.0-3.0 | Apixaban 2.5 mg BD | **7.70 (1.09, 76.4)** |

Significant results are in bold

CRNM bleeding

Table 5. Data used in analysis of CRNM bleeding

| **#** | **Treatment arm** | **No. of patients (N)** | **Number of patients with an event (n)** | **Relative risk**  **(95% CI)** |
| --- | --- | --- | --- | --- |
| AMPLIFY-EXT [[1](#_ENREF_1)] | Apixaban 2.5 mg BD | 840**^†^** | 25^‡^ | 1.29^#^  (0.72, 2.33) |
|  | Apixaban 5 mg BD | 811**^†^** | 34^‡^ | **1.82^#^**  **(1.05, 3.17)** |
|  | Placebo | 826 | 19^‡^ | - |
| ASPIRE [[5](#_ENREF_5)] | Aspirin 100 mg OD | 411^§^ | 6^¶^ | 3.00  (0.61, 14.78) |
|  | Placebo | 411^§^ | 2^¶^ |  |
| REMEDY [[4](#_ENREF_4)] | Dabigatran 150 mg BD | 1430^††^ | 67^§§^ | **0.56**  **(0.42, 0.74)** |
|  | Warfarin INR 2.0-3.0 | 1426^††^ | 120^¶¶^ |  |
| RESONATE [[4](#_ENREF_4)] | Dabigatran 150 mg BD | 684^‡‡^ | 34**^†††^** | **2.73**  **(1.43, 5.23)** |
|  | Placebo | 659^‡‡^ | 12^‡‡‡^ |  |
| EINSTEIN-EXT [[2](#_ENREF_2)] | Rivaroxaban 20 mg OD | 598^§§§^ | 32^¶¶¶^ | **4.51**  **(2.01, 10.14)** |
|  | Placebo | 590^§§§^ | 7^¶¶¶^ |  |
| WARFASA [[3](#_ENREF_3)] | Aspirin 100 mg OD | 205**^††††^** | 3^‡‡‡‡^ | 0.96  (0.20, 4.71) |
|  | Placebo | 197**^††††^** | 3^‡‡‡‡^ |  |

BD, twice daily; CI, confidence interval; INR, international normalized ratio; OD, once daily

Significant results in bold

# Relative risk represents treatment versus placebo.

†Patients included in safety analysis: data from patients during the time they were receiving treatment, defined as the time between administration of the first dose of a study drug and 48 hours after administration of the last dose.

‡Reported as ‘CRNM bleeding’ as reported in Table 2 of the publication [[1](#_ENREF_1)]

§All randomised patients

¶Reported as ‘CRNM bleeding’ in Table 2 of the publication [[5](#_ENREF_5)]

††The safety analysis was based on the treatment period and a 3-day washout period after the end of treatment.

‡‡The safety analysis included 684 patients who received dabigatran and 659 who received placebo. Three patients in the placebo group mistakenly received dabigatran throughout the study.

§§Calculated by subtracting major bleed (n=13) from ‘major or CRNM bleeding’ (n=80) as reported in Table 2 of the publication [[5](#_ENREF_5)]

¶¶Calculated by subtracting major bleed (n=25) from ‘major or CRNM bleeding’ (n=145) as reported in Table 2 of the publication [[5](#_ENREF_5)]

†††Calculated by subtracting major bleed (n=2) from ‘major or CRNM bleeding’ (n=36) as reported in Table 3 of the publication [[5](#_ENREF_5)]

‡‡‡Calculated by subtracting major bleed (n=0) from ‘major or CRNM bleeding’ (n=12) as reported in Table 3 of the publication [[5](#_ENREF_5)]

§§§Safety analyses included all patients who received the assigned study drug. Bleeding events were included in the analysis if they occurred during treatment or within 2 days after discontinuation of the study drug.

¶¶¶Reported as ‘CRNM bleeding’ in Table 4 of publication [[2](#_ENREF_2)] [some patients had more than 1 event]

††††All patients who received at least one dose of the study drug.

‡‡‡‡Reported as ‘CRNM bleeding’ in Table 2 of publication [[3](#_ENREF_3)]

Table 6. Relative risk fixed effects NMA results for CRNM bleeding (treatment vs. comparator)

| **Treatment** | **Comparator** | **Relative risk (95% CrI) (median)** |
| --- | --- | --- |
| Warfarin INR 2.0-3.0 | Apixaban 2.5 mg BD | **3.84 (1.55, 9.38)** |

BD, twice daily; CrI, credible interval; INR, international normalized ratio; OD, once daily

Significant results are in bold

Treatment Discontinuation

Table 7. Data used in analysis of overall treatment discontinuation

| **Study** | **Treatment arm** | **No. of patients (N)** | **Number of patients with an event (n)** | **Relative risk**  **(95% CI)** |
| --- | --- | --- | --- | --- |
| AMPLIFY-EXT [[1](#_ENREF_1)] | Apixaban 2.5 mg BD | 840^†^ | 114^‡^ | **0.60^#^**  **(0.48, 0.74)** |
|  | Apixaban 5 mg BD | 813^†^ | 129^‡^ | **0.70^#^**  **(0.57, 0.86)** |
|  | Placebo | 829^†^ | 188^‡^ | - |
| EINSTEIN-EXT [[2](#_ENREF_2)] | Rivaroxaban 20 mg OD | 602^†^ | 76^§^ | 0.81  (0.61, 1.07) |
|  | Placebo | 594^†^ | 93^§^ |  |
| LAFIT [[6](#_ENREF_6)] | Warfarin INR 2.0-3.0 | 79^¶^ | 14^††^ | 1.13  (0.57, 2.25) |
|  | Placebo | 83^¶^ | 13^††^ |  |
| PREVENT [[10](#_ENREF_10)] | Warfarin INR 1.5-1.9 | 255^¶^ | 64^‡‡^ | 1.13  (0.83, 1.55) |
|  | Placebo | 253^¶^ | 56^‡‡^ |  |
| REMEDY [[4](#_ENREF_4)] | Dabigatran 150 mg BD | 1430^§§^ | 276^¶¶^ | 0.98  (0.84, 1.14) |
|  | Warfarin INR 2.0-3.0 | 1426^§§^ | 281^¶¶^ |  |
| RESONATE [[4](#_ENREF_4)] | Dabigatran 150 mg BD | 681^§§^ | 71^¶¶^ | **0.70**  **(0.52, 0.93)** |
|  | Placebo | 662^§§^ | 99^¶¶^ |  |
| WARFASA [[3](#_ENREF_3)] | Aspirin 100 mg OD | 205^†††^ | 16^‡‡‡^ | 1.03  (0.52, 2.02) |
|  | Placebo | 197^†††^ | 15^‡‡‡^ |  |
| ASPIRE [[5](#_ENREF_5)] | Aspirin 100 mg OD | 411^¶^ | 117^§§§^ | 0.89  (0.72, 1.09) |
|  | Placebo | 411^¶^ | 132^§§§^ |  |
| ELATE [[7](#_ENREF_7)] | Warfarin INR 1.5-1.9 | 369^¶^ | 84^¶¶¶^ | **1.45**  **(1.07, 1.96)** |
|  | Warfarin INR 2.0-3.0 | 369^¶^ | 58^¶¶¶^ |  |

BD, twice daily; CI, confidence interval; INR, international normalized ratio; OD, once daily

Significant results are in bold

# Relative risk represents treatment versus placebo.

†Patients included in ITT analysis

‡Reported as ‘prematurely discontinued study treatment’ as reported in Figure 1 of the publication [[1](#_ENREF_1)]

§Reported as ‘premature discontinuation of treatment’ in Table 2 of publication [[2](#_ENREF_2)]

¶All randomised patients

††Reported as ‘premature discontinuation of study drug before the completion of follow up’ on p.903 of the publication [[6](#_ENREF_6)]

‡‡Reported as ‘discontinuation of study drug before the completion of follow up’ on p.1431 of the publication [[10](#_ENREF_10)].

§§Modified ITT: excluding patients who did not receive any dose of the study drug

¶¶Reported as ‘study drug stopped early’ on p.712 of publication [[4](#_ENREF_4)]

†††Modified ITT population: all patients who received at least one dose of the assigned study drug post randomisation.

‡‡‡Reported as ‘premature discontinuation of study drug’ on p.1961 of publication [[3](#_ENREF_3)]

§§§Reported as ‘total discontinuations’ in supplementary appendix Table 2 [[5](#_ENREF_5)]

¶¶¶Reported as ‘permanent discontinuation of double-blind treatment’ on p.633 of the publication [[7](#_ENREF_7)]

Table 8. Relative risk fixed effects NMA results for overall treatment discontinuation (treatment vs. comparator)

| **Treatment** | **Comparator** | **Relative risk (95% CrI) (median)** |
| --- | --- | --- |
| Warfarin INR 2.0-3.0 | Apixaban 2.5 mg BD | 1.33 (0.95, 1.87) |

BD, twice daily; CrI, credible interval; INR, international normalized ratio; OD, once daily

References

1. Agnelli G, Buller HR, Cohen A, Curto M, Gallus AS, Johnson M et al. Apixaban for extended treatment of venous thromboembolism. N Engl J Med. 2013;368(8):699-708.

2. Investigators E, Bauersachs R, Berkowitz SD, Brenner B, Buller HR, Decousus H et al. Oral rivaroxaban for symptomatic venous thromboembolism. N Engl J Med. 2010;363(26):2499-510.

3. Becattini C, Agnelli G, Schenone A, Eichinger S, Bucherini E, Silingardi M et al. Aspirin for preventing the recurrence of venous thromboembolism. N Engl J Med. 2012;366(21):1959-67.

4. Schulman S KC, Kakkar AK, Schellong S, Eriksson H, Baanstra D, Kvamme, AM FJ, Mismetti P, Goldhaber SZ;. RE-MEDY Trial Investigators; RE-SONATE Trial Investigators. Extended use of dabigatran, warfarin, or placebo in venous thromboembolism. N Engl J Med. 2013;368(8):709-18.

5. Brighton TA, Eikelboom JW, Mann K, Mister R, Gallus A, Ockelford P et al. Low-dose aspirin for preventing recurrent venous thromboembolism. N Engl J Med. 2012;367(21):1979-87.

6. Kearon C, Gent M, Hirsh J, Weitz J, Kovacs MJ, Anderson DR et al. A comparison of three months of anticoagulation with extended anticoagulation for a first episode of idiopathic venous thromboembolism. N Engl J Med. 1999;340(12):901-7.

7. Kearon C, Ginsberg JS, Kovacs MJ, Anderson DR, Wells P, Julian JA et al. Comparison of low-intensity warfarin therapy with conventional-intensity warfarin therapy for long-term prevention of recurrent venous thromboembolism. N Engl J Med. 2003;349(7):631-9.

8. Agnelli G, Prandoni P, Santamaria MG, Bagatella P, Iorio A, Bazzan M et al. Three months versus one year of oral anticoagulant therapy for idiopathic deep venous thrombosis. Warfarin Optimal Duration Italian Trial Investigators. N Engl J Med. 2001;345(3):165-9.

9. Agnelli G, Prandoni P, Becattini C, Silingardi M, Taliani MR, Miccio M et al. Extended oral anticoagulant therapy after a first episode of pulmonary embolism. Ann Intern Med. 2003;139(1):19-25.

10. Ridker PM, Goldhaber SZ, Danielson E, Rosenberg Y, Eby CS, Deitcher SR et al. Long-term, low-intensity warfarin therapy for the prevention of recurrent venous thromboembolism. N Engl J Med. 2003;348(15):1425-34.

11. Castellucci LA, Cameron C, Le Gal G, Rodger MA, Coyle D, Wells PS et al. Efficacy and safety outcomes of oral anticoagulants and antiplatelet drugs in the secondary prevention of venous thromboembolism: systematic review and network meta-analysis. BMJ. 2013;347:f5133.

12. Dias S, Welton, N.J., Sutton, A.J. & Ades, A.E. NICE DSU Technical Support Document 2: A Generalised Linear Modelling Framework for Pairwise and Network Meta-Analysis of Randomised Controlled Trials. wwwnicedsuorguk. 2011.

13. Higgins JPT, Green S. Cochrane Handbook for Systematic Reviews of Interventions. Version 510. 2011;Available at <http://handbook.cochrane.org/>.

14. Jansen JP, Fleurence R, Devine B, Itzler R, Barrett A, Hawkins N et al. Interpreting indirect treatment comparisons and network meta-analysis for health-care decision making: report of the ISPOR Task Force on Indirect Treatment Comparisons Good Research Practices: part 1. Value Health. 2011;14(4):417-28.

15. Spiegelhalter DJ. Bayesian measures of model complexity and fit. Journal of the Royal Statistical Society: Series B (Statistical Methodology). 2002;64(4):583-639.
